# Testing a Multicomponent Training Designed to Improve Sprint, Agility and Decision-Making in Elite Basketball Players

**DOI:** 10.3390/brainsci13070984

**Published:** 2023-06-22

**Authors:** Stefania Lucia, Mattia Digno, Iker Madinabeitia, Francesco Di Russo

**Affiliations:** 1 Department of Movement, Human and Health Sciences, University of Rome “Foro Italico”, 00135 Rome, Italy; francesco.dirusso@uniroma4.it; 2 Stella Azzurra Basketball Academy, 00191 Rome, Italy; mattiadigno@gmail.com; 3 Department of Physical Education and Sport, University of Granada, 18071 Granada, Spain; ikermadi@ugr.es; 4 Santa Lucia Foundation IRCCS, 00179 Rome, Italy

**Keywords:** cognitive-motor dual-task training, multi-component training, electroencephalographic (EEG), decision-making, decision speed

## Abstract

This study tested if, in elite basketball players’ training, the integration of a cognitive component within a multi-component training (MCT) could be more effective than an MCT with motor components only to improve both physical and cognitive skills. To this purpose, we designed an MCT focussed on sprint and agility incorporating a cognitive-motor dual-task training (CMDT) focussed on decision-making speed. Specific tests on sprint, agility and decision-making, and event-related potential (ERP) during the latter test were evaluated before and after the intervention. Thirty elite basketball players were recruited and divided into an experimental group executing CMDT integrated into the MCT and a control group performing the motor MCT (without cognitive components). The MCT with CMDT session was performed by four athletes simultaneously that executed different circuits. One circuit was the CMDT which was realized using interactive devices. Results on physical performance showed that only the experimental group improved in sprint and agility and also shortened response time in the decision-making test. At the neural level, the experimental group only shows an increase in the P3 ERP component, which has been associated with a series of post-perceptual cognitive functions, including decision-making. In conclusion, CMDT implemented within an MCT, likely stimulating more than physical training cortical plasticity, could be more effective than a motor MCT alone in improving the physical and cognitive skills of elite basketball players in five weeks only.

## 1. Introduction

Selecting the appropriate training is fundamental for reaching peak performance in any sport. In open-skill sports, the coordination of technical, physical, and mental components to be trained may represent a real challenge for coaches and sport psychologists. Multi-component training (MCT) may help improve in the same training session physical fitness, playing technique, cognitive performance, and consequently, the sport performance of athletes. In the same training session, MCT usually incorporates two or more exercise components such as agility, balance, speed, strength, or technique. MCT is usually used in athletes as warmup training to prevent injuries [1] but was hardly used inside training sessions [2,3], and the use of cognitive components was never considered. There is also evidence that in basketball players, MCT can improve anaerobic power and capacity, agility, and vertical jump height [4].

Another way to help coaches and sports psychologists train technical, physical, and mental components in a single session could be using cognitive-motor dual-task training (CMDT), in which cognitive and motor skills are trained simultaneously. CMDT is proven to be more effective than cognitive and motor training alone in improving both motor and cognitive performance [5]. CMDT can be designed to train specific motor and cognitive functions and has been successfully applied to improve dribbling performance in a professional basketball player [6,7,8]. CMDT uses technological devices to pace the athlete’s exercise and give immediate feedback about performance [9] and may use visuomotor response tasks during exercise programs [10,11].

Thus, based on these considerations, we here evaluated an MCT focused on sprint and agility incorporated into CMDT on decision-making speed that has not yet been studied in the literature.

In addition, to study the neural correlate of the possible CMDT effects, the event-related potentials (ERPs) method was used to analyze the brain activity associated with decision-making processes during the go/no-go task. To this aim, the P3 ERP component was considered because it is widely recognized to reflect cognitive processes associated with post-perceptual processing, such as stimulus evaluation and decision-making [12,13,14,15]. Decision-making is the cognitive process of controlling for the presence of task-relevant stimuli and mapping these onto appropriate responses [16]. The P3, with a medial centro-parietal scalp distribution, is evoked by stimuli evaluation in tasks that require any form of action, such as a response to targets [12,17,18,19]. The P3 amplitude positively correlated to response time (for normative data, see [20]) and with greater confidence in the decision [21]. Considering that the P3 amplitude facilitates responding based on the decision we expect after the MCT combined with CMDT, larger P3 and faster response time in the experimental group only.

In the present study, we sought to prove if the use of CMDT within an MCT could be more effective than a motor MCT alone in improving the physical and cognitive skills of elite basketball players. To this aim, we designed an MCT focussed on sprint and agility with the addition of a CMDT focussed on decision-making speed.

## 2. Materials and Methods

### 2.1. Participants

Thirty young basketball players (15 females and 15 males with ages ranging from 15 to 17 years) were recruited for the study. All athletes were part of the “Stella Azzurra Basketball Academy Rome” sport society. The sample size was determined using the G*power 3.1.9.7 statistical program for 2 × 2 within-between repeated measure test. We set the effect size f at 0.281 based on the mean significant partial eta squared effect size obtained in [6,7,8], which used a similar statistical design. The α level was set at 0.05, and the desired power (1−β error probability) at 0.95 [22]. The inclusion criteria were: no injuries prior to preliminary testing, being actively involved in competitive basketball for at least 6 years, and competing regularly in under-18 championships. Both parents of all participants gave their informed consent before participating in this study in accordance with the Declaration of Helsinki after approval by the local ethical committee of the University of Rome “Foro Italico”.

### 2.2. Study Design

In order to evaluate the efficacy of the proposed training, a randomized-control trial was applied to elite basketball players using the 10 m sprint and change of direction (COD) tests as sprint and agility outcomes. The Go/No-go task was used to evaluate the decision-making speed. This task, in which participants produce motor responses to targets and do not respond to non-targets, is widely used to study the neural basis of motor response evaluation and execution [12].

**Figure 1:**
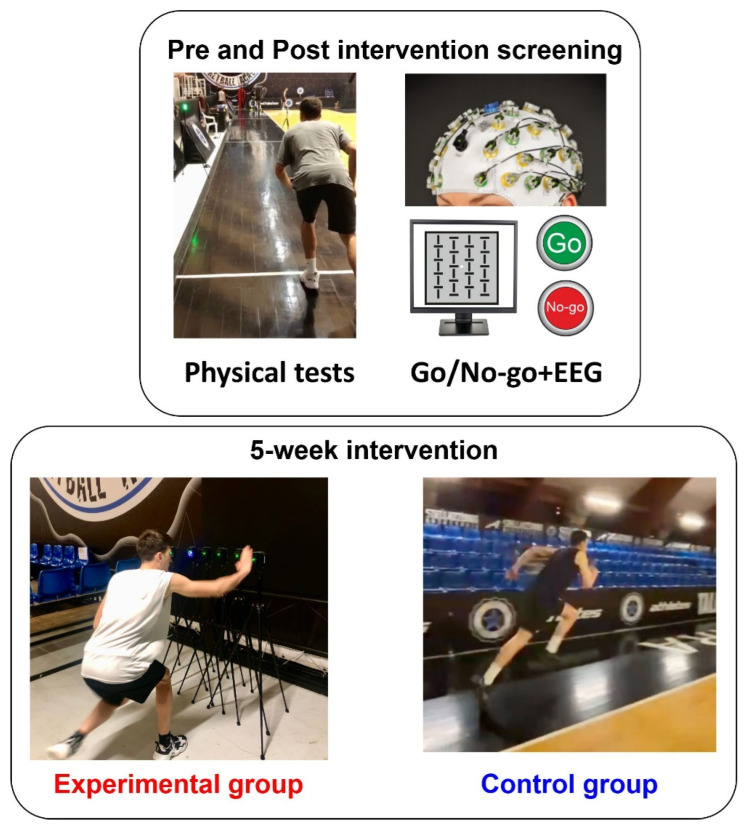
Experimental procedure.

A graphical representation of the experimental procedure is in Figure 1. Before and after the experimental protocol, all participants completed specific tests for sprint performance, COD (physical tests), and the Go/No-go for decision-making speed, which was performed during electroencephalographic (EEG) recording. Physical tests were executed two days before and after EEG recording. Cognitive tasks and EEG recordings were performed one day before and after EEG recording.

Participants were randomly assigned to two groups using a random table: 15 in the experimental group (Exp) and 15 in the control group (Con). Then, groups were balanced for sex to avoid potential sex-related bias, as found in [8]. Groups did not differ in age or in expertise. Regarding expertise, the McKinney classification [23] suggests an elite level.

### 2.3. Physical Tests

Two physical tests based on sprint and COD, fundamentals of basketball, were used to verify the treatment effects on performance. These tasks were based on linear speed capabilities. More specifically, the 10 m sprint test was used to measure acceleration and COD to test agility. These tests were conducted on the basketball court. Photocells were used to record completion time in seconds. In addition, one semaphore (Witty-SEM device described below) was placed in front of the athletes. The semaphore showed a countdown (e.g., 3-2-1), and after displaying, the number 1 became black. Then, with a random interval (1–5 s), the semaphore became green, indicating the athletes to start, in separate sessions, the sprint or the COD test. In the sprint test, athletes performed a linear 10 m run-up to the “stop” position signaled by the photocells. In the COD test, athletes were placed in front of the countdown semaphore, but additionally, the green starting signal was an arrow. Depending on the direction of the green arrow given, they had to sprint right or left for 5 m (marked by a cone), change direction, run back for 10 m up another cone, change direction again, and finally run for another 5 m back to their central starting position. All the exercises were repeated three times, and the best time for each athlete was taken into account. In order to verify the reliability of the test, the three repetitions were submitted intraclass correlation calculating the intraclass correlation coefficient (ICC) for two-way mixed effects. This analysis showed high correlations among repetition for both the pre (ICC = 0.78) and the post-test (ICC = 0.79), indicating good inter-rater reliability of the test.

### 2.4. Go/No-Go Task

This task was performed during EEG recording in a sound-attenuated, dimly little room. Athletes were comfortably seated in front of a computer screen at a distance of 114 cm from their eyes with a response pad positioned under their right index finger. A yellow fixation point (diameter 0.15 × 0.15°) on a black background was present in the center, and that never disappeared during the experimental session. Four visual stimuli (i.e., square configurations subtending 4 × 4° and consisting of vertical and/or horizontal bars) were randomly visualized for 250 ms with equal probability (*p* = 0.25); the stimulus–onset asynchrony varied from 1 to 2 s to prevent stimulus prediction and ERP overlaps with previous and following stimuli. Participants had to press a response button with the right index finger as soon as possible only when target stimuli (two out of four) resulted on the screen (*p* = 0.5) and to withhold the motor response when non-target stimuli compared (*p* = 0.5); time reaction and accuracy were equally emphasized by the experimenter. The order of presentation of the four targets or non-targets was randomized between runs. The duration was 2 min for each run with a pause interleaved. Ten runs were administered, allowing us to obtain 400 trials for each stimulus category in approximately 30–40 min, depending on the participant’s rest time during the experimental session. The response time (RT) for target stimuli was taken as an index of decision-making speed.

### 2.5. EEG Recording and Analysis

EEG data were recorded using a 64-channel EEG system (Brainamp™ amplifier) with active electrodes (Acticap™) and software (Recorder 1.2 and Analyzer 2.2.2), all by Brain Products GmbH (Munich, Germany). The electrodes were mounted according to the 10–10 international system and referenced to the average of the M1–M2 electrodes. The EEG was amplified, digitized at 250 Hz, band-pass filtered using a Butterworth zero-phase filter (0.01–40 Hz and 50 Hz notch filter; second order), and stored for offline analyses. The eye movements were controlled by electrooculogram (EOG) recorded by the third BrainAmp amplifier (ExG type) in bipolar modality. Horizontal EOG was recorded with an electrode pair over the left and right outer canthi of the eyes, while vertical EOG (VEOG) was recorded with an electrode pair below and above the left eye. Electrode impedances were kept below 5 KΩ. The corrections of blink and vertical eye movement artifacts were automatically signed by means of the independent component analysis. Data were then submitted to automatic artifact rejection, excluding EEG with amplitudes exceeding the threshold of ±70 µV. In order to evaluate the post-stimulus ERP activity, EEG was segmented into 1000 ms epochs, starting 100 ms before and ending 900 ms after stimulus onset and using the first 100 ms (−100/0 ms) as the baseline. Target and non-target trials were averaged separately. In order to select the intervals and electrodes to be taken into consideration in statistical analysis, the “collapsed localizer” method was used [24], in which a localizer ERP is obtained by collapsing (averaging) all experimental conditions. In order to identify the interval of analysis, the global field power (GFP) was calculated. The GFP describes the ERP spatial variability at each time point considering all scalp electrodes simultaneously, resulting in a reference-independent descriptor of the potential field. The 300–800 ms post-stimulus interval was used to identify the P3 component, and within this interval, the timeframe in which the GPF was larger than 80% of its maximum value was used for further analysis. This GFP approach selected one interval from 480 ms to 580 ms in which the mean amplitude was calculated in all conditions for statistical purposes. The electrodes with an amplitude larger than 80% of the maximum value in the intervals selected by the collapsed localizer were jointed in spatial pools and considered for statistical analysis. Two foci of activity were clearly present for P3: centroparietal distribution for target trials and a medial frontocentral distribution for non-target trials. The P3 was then represented by a pool for target trials containing CP1, CPz, CP2, P1, Pz, and P2 electrodes (centroparietal pool), and for non-target trials, P3 was represented by a pool containing C1, Cz, C2, FC1, FCz and FC2 electrodes (frontocentral pool).

### 2.6. CMDT Protocol

Both groups performed the same week program (e.g., five 3 h basketball training sessions and a match during the weekend). Included in their training, they performed two 30 min physical sessions on sprint and agility. For the Con, the physical session was an MCT, including four circuits repeated once, including sprint/agility and core-balance exercises. The Exp performed the same physical sessions with the same exercise circuits but simultaneously performed specific cognitive tasks. The intervention duration was five weeks.

**Figure 2:**
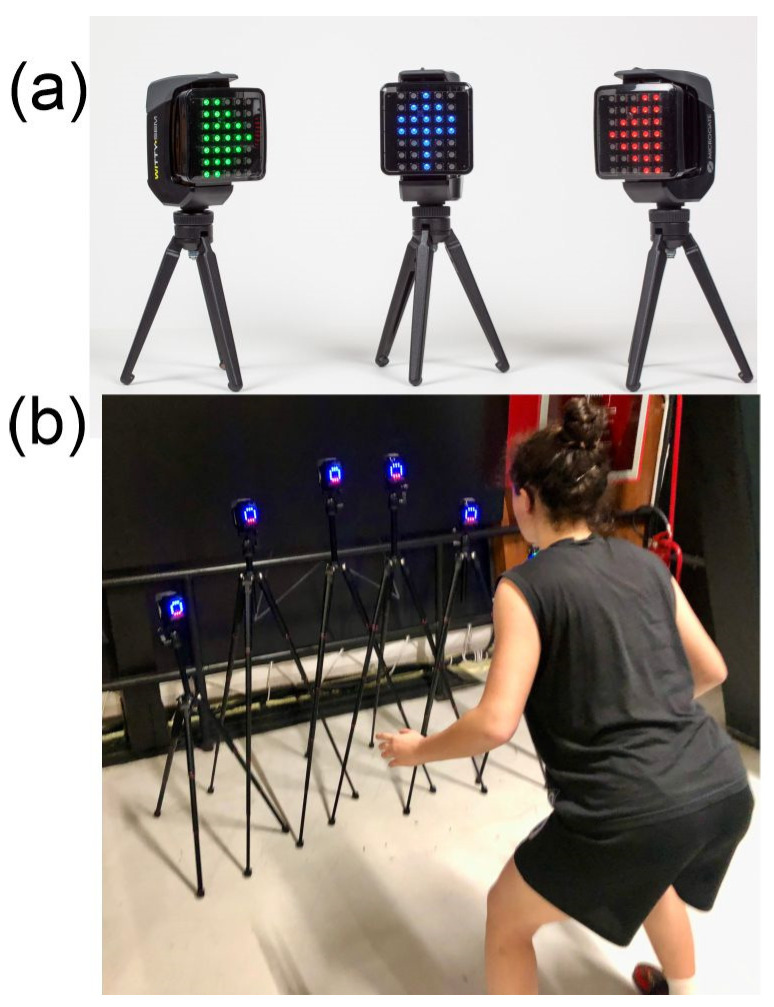
(**a**) Interactive display devices (Witty-SEM). (**b**) Video frame showing a moment of the cognitive-motor training.

The CMDT constituted the experimental treatment and was inserted concurrently with the sprint/agility tasks. The CMTD required the simultaneous execution of tasks requiring efficient working memory, fast decision-making, and response speed. The exercises were organized in short routines that alternated with an exercise and core stability. The CMDT was realized using six Witty-SEM devices (Microgate, Bolzano, Italy) that, with different heights, were positioned in front of athletes aligned or in a semicircle. Each Witty-SEM device was composed of a led screen displaying patterns of three colors, emitting sounds, and interacting with users thanks to proximity sensors. These devices are shown in Figure 2a. The CMDT consisted of four exercises requiring physical-specific skills such as sprint, speed, footwork, and CODs and, at the same time, required tasks such as discriminative responses, visual search, target matching, and number ordering, promoting cognitive functions including visual speed, working memory, and decision-making (Figure 2b). The task difficulty (exposure time, inter-stimulus interval, number of items) was adaptively modulated according to the athlete’s performance.

### 2.7. Statistical Analysis

The Shapiro-Wilk’s W test was performed for all measures, confirming their normal distributions. In order to test the assumption of homoscedasticity, the Levene test for equality of variance was performed, showing no violation of the sample homoscedasticity. After this preliminary testing, all measures (except for the P3) were submitted to 2 × 2 ANOVAs with Group (Exp vs. Con) and Intervention (Pre-test vs. Post-test) as factors. The P3 was submitted to 2 × 2 × 2 ANOVA with Trial (Go vs. No-go) as an additional factor. For significant comparisons, effect sizes were reported in terms of partial eta squared (η_p_^2^). For post-hoc comparisons, the Bonferroni correction was used to compensate for multiple comparisons. The overall alpha level was fixed at 0.05. All statistical analyses were performed using the Statistica 12.0 software (StatSoft Inc., Tulsa, OK, USA).

## 3. Results

### 3.1. Physical Tests

**Figure 3:**
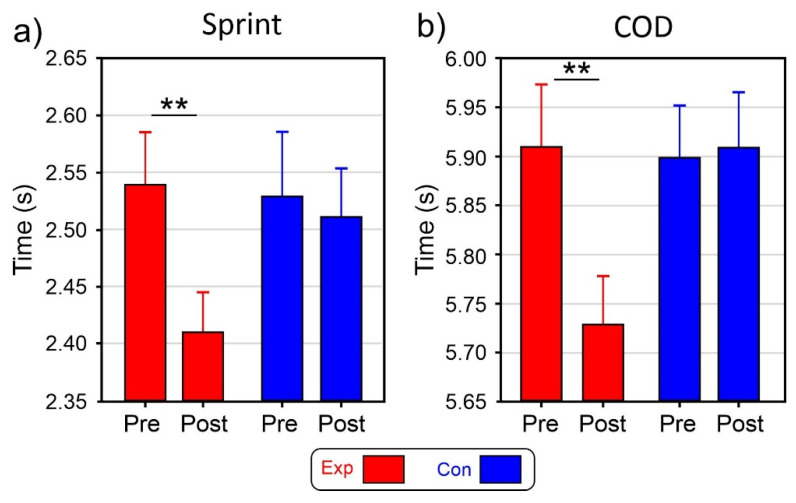
Physical test results. (**a**) 10 m sprint test of the experimental (Exp) and control (Con) groups and the pre (Pre) and post-test (post). (**b**) change of direction (COD) test completion time of the Exp and Con groups in the two tests. Vertica bars indicate 0.95 confidence intervals ** *p* < 0.01.

ANOVA on the sprint test indicated that the effect of the Group was not significant (F_1,28_ = 0.8, *p* = 0.394). The effect of the Intervention (F_1,28_ = 27.7, *p* < 0.001, η_p_^2^ = 0.497) and the interaction (F_1,28_ = 16.0, *p* < 0.001, η_p_^2^ = 0.363) were significant. Post-hoc comparisons showed that in the post-test, the completion time of the Exp (2.41 s SD = 0.11) was shorter (*p* < 0.001) than the Pre-test time (2.54 s SD = 0.10). In the Con, the difference between the Pre-test (2.53 s SD = 0.19) and the Post-test (2.51 s SD = 0.14) was not significant (Figure 3a).

ANOVA on the COD test indicated that the effect of the Group was not significant (F_1,28_ = 0.2, *p* = 0.652). The effect of the Intervention (F_1,28_ = 4.5, *p* = 0.036, η_p_^2^ = 0.148) and the interaction (F_1,28_ = 11.4, *p* = 0.002, η_p_^2^ = 0.289) were significant. Post-hoc comparisons showed that in the Exp, post-test, the completion time (5.73 s SD = 0.28) was shorter (*p* = 0.003) than the pre-test time (5.92 s SD = 0.34). In the Con, the difference between the Pre-test (5.90 s SD = 0.30) and the Post-test (5.91 s SD = 0.32) was not significant (Figure 3b).

### 3.2. Go/No-Go Task

ANOVA on the RT in the Go/No-go task showed that the Group effect was non-significant (F_1,28_ = 0.3, *p* = 0.428) while the Intervention effect (F_1,28_ = 21.3, *p* < 0.001, η_p_^2^ = 0.451) and the interaction (F_1,28_ = 4.9, *p* = 0.035, η_p_^2^ = 0.160) were significant. Post-hoc comparisons showed that the Exp Intervention only was effective (pre-test 477 ms SD = 53; post-test 431 ms SD = 47, *p* < 0.001), but not the control training (pre-test 478 ms SD = 56; post-test 461 ms SD = 48).

### 3.3. ERP Results

**Figure 4:**
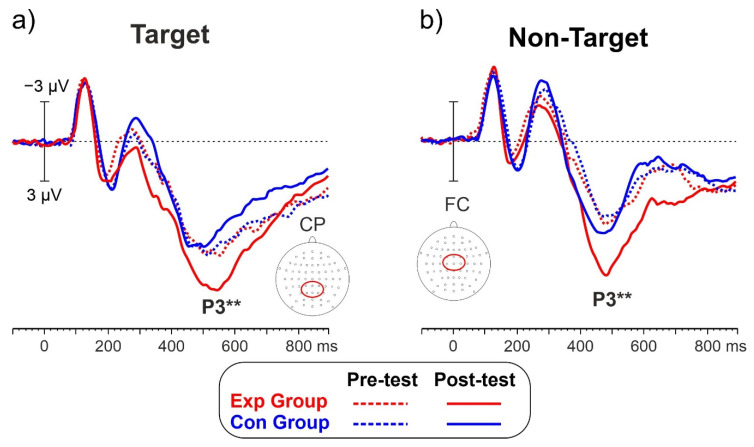
ERP waveforms (**a**) P3 for target trials with a centroparietal distribution (CP). (**b**) P3 for non-target trials with a medial frontocentral (FC) distribution. ** *p* < 0.01.

Figure 4 shows the ERP waveforms of the two groups before and after the intervention and for the target (Figure 4a) and non-target (Figure 4b) conditions. The P3 is labeled and had a mean peak latency of 463 ms with a centroparietal distribution for target trials and a medial frontocentral distribution for non-target trials.

**Figure 5:**
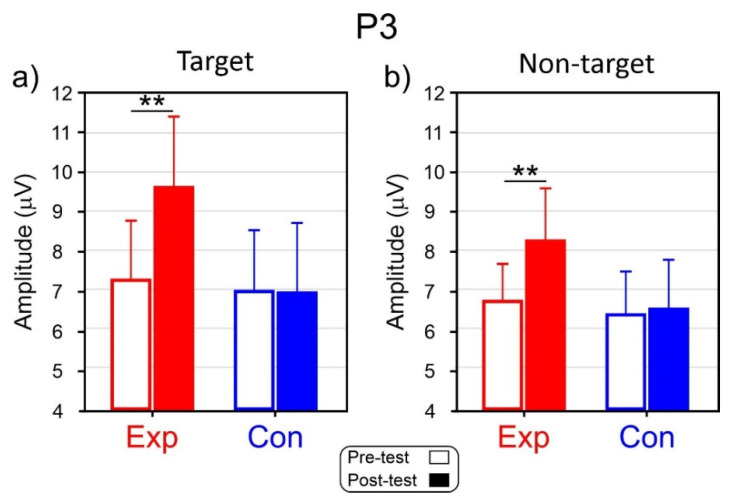
Mean amplitude of the P3. (**a**) Target condition in the experimental (Exp) and control groups. (**b**) Non-target condition in Exp and Con groups. Vertical bars indicate 0.95 confidence intervals ** *p* < 0.01.

The ANOVA on the P3 amplitude showed a non-significant effect of the Group factor (F_1,28_ = 2.6, *p* = 0.117). The effects of the Intervention (F_1,28_ = 5.9, *p* = 0.022, η_p_^2^ = 0.174) and of the Condition (F_1,28_ = 4.5, *p* = 0.042, η_p_^2^ = 0.139) were significant. The Treatment*Group interaction was also significant (F_1,28_ = 6.2, *p* = 0.021, η_p_^2^ = 0.175). Post-hoc comparisons showed that in the Exp, the P3 amplitude in the post-test (8.96 μV SD = 1.06) was larger (*p* = 0.007) than the amplitude in the pre-test (7.03 μV SD = 0.92). For the Con, the difference between the Pre-test (6.71 μV SD = 0.87) and the Post-test (6.74 μV SD = 0.89, *p* = 0.008) was not significant. The P3 amplitude (7.72 μV SD = 0.96) for the Go condition was larger (*p* = 0.042) than the P3 for the No-go condition (6.99 μV SD = 0.91). P3 amplitudes are shown in Figure 5 for both the target (Figure 5a) and non-target (Figure 5b) conditions.

## 4. Discussions

In the present study, we tested if the use of a novel CMDT implemented within an MCT could be more effective than a motor MCT alone in improving the physical and cognitive skills of elite basketball players in just five weeks. We also aimed to confirm if the previously observed CMDT efficacy on sport performance could also be extended to physical performance such as sprint and agility. In addition, the effect on decision speed and the neural correlates of post-sensory decision-making processes were also investigated.

Results showed that the experimental MCT (with CMDT) intervention strongly improved both sprint and agility, while the standard MCT (without CMDT) was ineffective. This evidence shows that the CMDT could be effective, not only on sport-specific accomplishment, as previously shown for the dribbling fundamental [6,7,8], but also on general physical performance. The fact that the experimental intervention was effective for both sprint and agility implies that it may have a broad range of benefits for physical performance. Sprinting and agility are important components of many sports and physical activities, and improving these abilities could lead to better performance in a variety of contexts. These findings support the existing literature on the benefits of these two skills. Sprinting and agility benefit athletic performance by, for example, improving attacking or defending abilities during a game [25]. At the cognitive level, the experimental treatment improved response speed, likely increasing post-perceptual attentional allocation and decision-making processes. The neural basis of this effect has been identified in the increase in the P3 ERP component, which has been associated with a series of post-perceptual cognitive functions, including decision-making [26]. Results confirm that ERP is a valid tool to empirically constrain the neural chronometry of perceptual decision-making [27].

This result could be explained by the fact that a key aspect of the experimental training was anticipation and quick identification of task-relevant stimuli in variable situations. These skills are particularly important in sport training, as highlighted in the “Situation Model of Anticipated Response consequences of Tactical” (SMART) training [28]. In this training model, the decision-making processes in sports are affected by implicit and explicit learning and can be trained by also proposing video feedback on game situations. The SMART model includes specific predictions based on the complexity of a situation, which can be manifested in the number of choice options, the visual information available, and the speed with which the decision needs to be made. Indeed, the experimental protocols in which the athletes are involved include these elements; in fact, it is a complex training in which motor exercises and cognitive tasks must be handled simultaneously and in a circuit, and the number of choice options varies from both motor and cognitive perspective. Regarding the decision-making speed, it is manipulated mainly by the cognitive exercise because the system proposes a time-adaptive exercise where the more athletes respond correctly, the faster and more challenging exercises become. Moreover, the combination of MCT and CMDT forced the athletes to move quickly from one circuit to the next, further stimulating decision speed.

In all tests, the control treatment was ineffective likely because physical training requires months to reach substantial results [29,30], especially in basketball [31], and the short training duration (five weeks) was not enough. As previously shown [6,7,8], this negative result concurs to confirm the strength and efficiency of the training proposed here, which could allow professional players appreciable results in just a few weeks.

Future studies could consider different sport disciplines to generalize the results, follow-up analyses to monitor the effect’s duration over time, and could evaluate whether a longer training covering an entire season could be even more effective.

## 5. Conclusions

Concluding CMDT implemented within an MCT could be more effective than a motor MCT alone in improving the physical and cognitive skills of elite basketball players in just five weeks. The results refute the idea that, in CMDT, cognitive exercise is just a distraction to motor exercise [32], so using cognitive exercise in an MCT, strength and conditioning coaches could use this innovative training to obtain more benefits than standard MCT in less time.

The combination of motor and cognitive exercise may boost physical training effects in any athlete, likely because the effort to adapt and change behavior in response to complex and enriched experiences may induce neural network plastic changes by the acquisition and execution of multiple motor and cognitive skills during training.

## Data Availability

Data are available from the corresponding author upon request.

## References

[1] Brunner R., Friesenbichler B., Casartelli N.C., Bizzini M., Maffiuletti N.A., Niedermann K. (2019). Effectiveness of multicomponent lower extremity injury prevention programmes in team-sport athletes: An umbrella review. Br. J. Sport. Med..

[2] Francino L., Villarroel B., Valdés-Badilla P., Ramirez-Campillo R., Martín E.B.-S., Ojeda-Aravena A., Aedo-Muñoz E., Pardo-Tamayo C., Herrera-Valenzuela T. (2022). Effect of a Six Week In-Season Training Program on Wrestling-Specific Competitive Performance. Int. J. Environ. Res. Public Health.

[3] Herrera-Valenzuela T., Ivaldes-Badilla P., Franchini E., Santos J., Ramirez-Campillo R., García-Hermoso A., Durán S., Castañeda-Gómez J.P. (2016). Effects of multi-component training on the physical fitness of young taekwondo athletes. Ido Movement for Culture. J. Martial Arts Anthropol..

[4] Brijwasi T., Borkar P. (2022). To study the effect of sports specific training program on selective physical and physiological variables in basketball players. Int. J. Phys. Educ. Sport. Health.

[5] Romeas T., Guldner A., Faubert J. (2016). 3D-Multiple Object Tracking training task improves passing decision-making accuracy in soccer players. Psychol. Sport Exerc..

[6] Lucia S., Bianco V., Boccacci L., Di Russo F. (2022). Effects of a Cognitive-Motor Training on Anticipatory Brain Functions and Sport Performance in Semi-Elite Basketball Players. Brain Sci..

[7] Lucia S., Bianco V., Di Russo F. (2022). Specific effect of a cognitive-motor dual-task training on sport performance and brain processing associated with decision-making in semi-elite basketball players. Psychol. Sport Exerc..

[8] Lucia S., Aydin M., Di Russo F. (2023). Sex Differences in Cognitive-Motor Dual-Task Training Effects and in Brain Processing of Semi-Elite Basketball Players. Brain Sci..

[9] Weart A.N., Miller E.M., Freisinger G.M., Johnson M.R., Goss D.L. (2020). Agreement between the OptoGait and instrumented treadmill system for the quantification of spatiotemporal treadmill running parameters. Front. Sport. Act. Living.

[10] Badau D., Badau A. (2022). Optimizing Reaction Time in Relation to Manual and Foot Laterality in Children Using the Fitlight Technological Systems. Sensors.

[11] Forni F., Farinini E., Leardi R., Rinaldo A. (2022). Effects of visual training on motor performance in young tennis players using FitLight trainer. J. Sport. Med. Phys. Fit..

[12] Di Russo F., Berchicci M., Bianco V., Perri R.L., Pitzalis S., Quinzi F., Spinelli D. (2019). Normative event-related potentials from sensory and cognitive tasks reveal occipital and frontal activities prior and following visual events. Neuroimage.

[13] Andreass J.L. (2000). Event-Related Slow Brain Potentials and Behavior. Psychophysiology: Human Behavior and Physiological Response.

[14] Nieuwenhuis S., Aston-Jones G., Cohen J.D. (2005). Decision making, the P3, and the locus coeruleus–norepinephrine system. Psychol. Bull..

[15] Rohrbaugh J.W., Donchin E., Eriksen C.W. (1974). Decision making and the P300 component of the cortical evoked response. Percept. Psychophys..

[16] Gold J.I., Shadlen M.N. (2001). Neural computations that underlie decisions about sensory stimuli. Trends Cogn. Sci..

[17] Kok A. (2001). On the utility of P3 amplitude as a measure of processing capacity. Psychophysiology.

[18] Polich J. (2007). Updating P300: An integrative theory of P3a and P3b. Clin. Neurophysiol..

[19] Verleger R. (2020). Effects of relevance and response frequency on P3b amplitudes: Review of findings and comparison of hypotheses about the process reflected by P3b. Psychophysiology.

[20] Conroy M.A., Polich J. (2007). Normative variation of P3a and P3b from a large sample: Gender, topography, and response time. J. Psychophysiol..

[21] Hillyard S.A., Squires K.C., Bauer J.W., Lindsay P.H. (1971). Evoked potential correlates of auditory signal detection. Science.

[22] Faul F., Erdfelder E., Buchner A., Lang A.-G. (2009). Statistical power analyses using G*Power 3.1: Tests for correlation and regression analyses. Behav. Res. Methods.

[23] McKinney J., Velghe J., Fee J., Isserow S., Drezner J.A. (2019). Defining athletes and exercisers. Am. J. Cardiol..

[24] Luck S.J., Gaspelin N. (2017). How to get statistically significant effects in any ERP experiment (and why you shouldn’t). Psychophysiology.

[25] Young W.B., Dawson B., Henry G.J. (2015). Agility and change-of-direction speed are independent skills: Implications for training for agility in invasion sports. Int. J. Sport. Sci. Coach..

[26] Filipović S.R., Jahanshahi M., Rothwell J.C. (2000). Cortical potentials related to the nogo decision. Exp. Brain Res..

[27] Kopp B., Tabeling S., Moschner C., Wessel K. (2007). Temporal dynamics of selective attention and conflict resolution during cross-dimensional go-nogo decisions. BMC Neurosci..

[28] Raab M. (2003). Decision making in sports: Influence of complexity on implicit and explicit learning. Int. J. Sport Exerc. Psychol..

[29] Taskin H. (2009). Effect of circuit training on the sprint-agility and anaerobic endurance. J. Strength Cond. Res..

[30] Hammami M., Negra Y., Shephard R.J., Chelly M.S. (2017). The effect of standard strength vs. contrast strength training on the development of sprint, agility, repeated change of direction, and jump in junior male soccer players. J. Strength Cond. Res..

[31] Cengizel E., Cengizel Ç.Ö., Öz E. (2020). Effects of 4-month basketball training on speed, agility and jumping in youth basketball players. Afr. Educ. Res. J..

[32] Laessoe U., Hoeck H.C., Simonsen O., Voigt M. (2008). Residual attentional capacity amongst young and elderly during dual and triple task walking. Hum. Mov. Sci..

